# Spatially Controlled Surface Modification of Porous Silicon for Sustained Drug Delivery Applications

**DOI:** 10.1038/s41598-018-37750-w

**Published:** 2019-02-04

**Authors:** De-Xiang Zhang, Chiaki Yoshikawa, Nicholas G. Welch, Paul Pasic, Helmut Thissen, Nicolas H. Voelcker

**Affiliations:** 1 Drug Delivery, Disposition and Dynamics, Monash Institute of Pharmaceutical Sciences, Monash University, Parkville, Victoria 3052 Australia; 2 Commonwealth Scientific and Industrial Research Organisation (CSIRO) Manufacturing, Clayton, Victoria 3168 Australia; 3 International Centre for Materials Nanoarchitectonics, National Institute for Materials Science, 1-2-1, Sengen, Tsukuba, Ibaraki 305-0047 Japan; 4 Melbourne Centre for Nanofabrication, Victorian Node of Australian National Fabrication Facility, Clayton, Victoria, 3168 Australia

## Abstract

A new and facile approach to selectively functionalize the internal and external surfaces of porous silicon (pSi) for drug delivery applications is reported. To provide a surface that is suitable for sustained drug release of the hydrophobic cancer chemotherapy drug camptothecin (CPT), the internal surfaces of pSi films were first modified with 1-dodecene. To further modify the external surface of the pSi samples, an interlayer was applied by silanization with (3-aminopropyl)triethoxysilane (APTES) following air plasma treatment. In addition, copolymers of *N*-(2-hydroxypropyl) acrylamide (HPAm) and *N*-benzophenone acrylamide (BPAm) were grafted onto the external pSi surfaces by spin-coating and UV crosslinking. Each modification step was verified using attenuated total reflection-Fourier transform infrared (ATR-FTIR) spectroscopy, water contact angle (WCA) measurements, X-ray photoelectron spectroscopy (XPS) and scanning electron microscopy (SEM). In order to confirm that the air plasma treatment and silanization step only occurred on the top surface of pSi samples, confocal microscopy was employed after fluorescein isothiocyanate (FITC) conjugation. Drug release studies carried out over 17 h in PBS demonstrated that the modified pSi reservoirs released CPT continuously, while showing excellent stability. Furthermore, protein adsorption and cell attachment studies demonstrated the ability of the graft polymer layer to reduce both significantly. In combination with the biocompatible pSi substrate material, the facile modification strategy described in this study provides access to new multifunctional drug delivery systems (DDS) for applications in cancer therapy.

## Introduction

Nanostructured materials have emerged as promising candidates for drug delivery, especially for cancer treatment^1^. Apart from the conventional polymer- and lipid-based drug delivery systems (DDS) and other inorganic nanomaterials^2–5^, porous Si (pSi) is an attractive material for nanomedicine applications due to its remarkable properties such as the large surface area (up to 800 m^2^·g^−1^)^6^, biocompatibility^7–9^ and biodegradability^10–12^ while retaining drug bioactivity^13^. In addition, nanostructured pSi has shown unique optical^14^ and luminescent properties^11,15^, which are conducive to self-reporting drug loading and release. pSi can be fabricated into films^6,16–20^, microparticles^21–23^ or nanoparticles^11,24–27^. Drug loading capacities and release kinetics highly depend on the surface area and surface chemistry of pSi materials^28,29^. And not surprisingly, hydrophobic drugs load more efficiently into hydrophobic pores^17,28^. However, it can be difficult to wet drug reservoirs with hydrophobic outer surfaces when used under physiological conditions. Therefore, the decoration of pSi internal and external surfaces with distinct properties is highly sought after^30^.

Selective modification on pSi outer surfaces was first reported by Cunin and coworkers^31^. Here, flat silicon was thermally hydrosilylated with hydrocarbons followed by electrochemical etching. The organic layer remained at least partially on the outer surface while fresh pores were presented, leaving inner surfaces available for further modification. For inner surface modification, several anodization and silanization cycles were applied on aluminium oxide membranes, resulting in spatially controlled surface modifications^32,33^. Kilian *et al*. presented a differential functionalization on exterior and interior pSi surfaces which relied on the combination of surface tension and capillarity^30^. The entire surface was first modified with hydrophobic 10-succinimidylundecenoate which could effectively repel water from penetrating into the inner pores, resulting in peptide conjugation only occurring on the outer surfaces. In contrast, using organic solvents, different reactive solutes were attached to the inner pSi surfaces. Similarly, Wu and Sailor used an inert liquid as a “mask” to protect the inner pores from being attacked by hydrofluoric acid solution while selectively functionalizing the external pSi surface^34^. More recently, a three step functionalization procedure consisting of two hydrosilylation reactions separated by the selective etching of the external surface was described. This procedure provides the outer pSi surface with hydrophilic groups while restricting hydrophobization to the inner pore walls, which was demonstrated by angle-resolved X-ray photoelectron spectroscopy (XPS) using pSi macro pores^35^. A less pore size-dependent procedure was also reported, with a light-assisted hydrosilylation reaction yielding a discrete surface chemistry on pSi layers, where the depth of chemical modification depends on the wavelength of light used in the procedure^36^. However, pSi exterior surface modification with polymers, in particular anti-fouling polymers, while decorating interior pore walls with hydrophobic species has not been demonstrated so far.

Exploring a DDS composed of pSi and a polymer with antifouling properties is desirable as the fate of biomaterials can be adversely affected by non-specific protein adsorption and cell attachment when applied *in vivo*. A range of different polymeric coatings have been under investigation, including poly(ethylene glycol) (PEG) based polymers, zwitterionic polymers and acrylate or acrylamide polymers such as *N*-(2-hydroxypropyl) methacrylamide (HPMAm) polymer^37,38^. The monomer *N*-(2-hydroxypropyl) acrylamide (HPAm), an analogue of HPMAm was first introduced by Fairbanks *et al*.^39^. In this study, HPAm polymer hydrogels were prepared by reversible addition-fragmentation chain transfer (RAFT)-mediated polymerization, with much faster polymerization kinetics compared to HPMAm. In terms of reducing protein adsorption and cellular attachment, HPAm hydrogels performed equivalently or better than materials made from HPMAm and PEG. Herein, in order to improve control over the drug release behavior while also imparting antifouling properties to nanostructured pSi, HPAm polymer grafting was applied. To achieve this, pSi films were first hydrosilylated with hydrocarbon chains, then treated with air plasma, which was expected to remove the organic species on the top surface of pSi and also leave behind a thin oxide layer. This was followed by silanization and then loading with the hydrophobic drug camptothecin (CPT). Finally, a cross-linked HPAm based polymer was grafted to the pSi outer surface via UV irradiation. CPT possessing intrinsic fluorescence properties was chosen as a model drug, since it has low solubility in aqueous solutions^19^. The CPT was loaded into the pores of the pSi matrix by physical adsorption and the release kinetics was adjusted by both hydrophobic inner pore wall modification and the grafting of an outer polymer barrier. Protein adsorption and cell attachment experiments were conducted to evaluate the antifouling properties of the outer polymer coating on pSi surfaces.

## Results and Discussion

### Sample preparation and characterization

**Figure 1:**
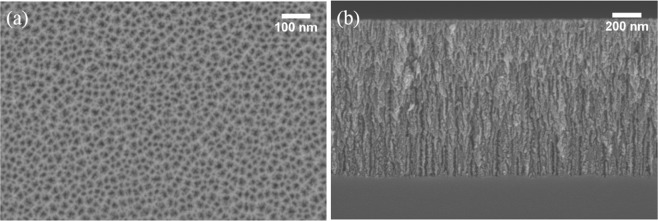
Typical SEM images of pSi used in this study with (**a**) top-view, (**b**) cross-sectional view.

**Figure 2:**
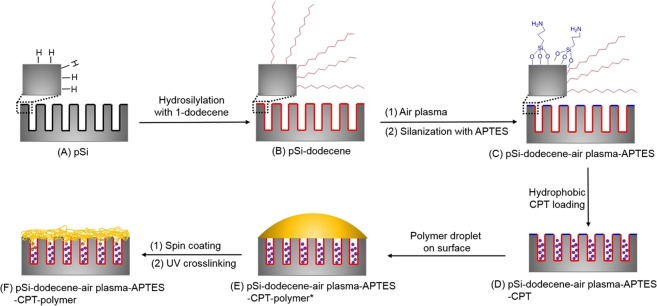
Schematic diagram showing the differential functionalization of interior and exterior surfaces of pSi films to allow improved camptothecin (CPT) drug loading and release.

pSi layers were fabricated using an electrochemical anodization process with a hydrofluoric acid/ ethanol mixture as the electrolyte. The pSi layers had an average pore size of 15 nm and depth of 1 µm as determined by SEM (Fig. 1). As shown in Fig. 2, pSi layers were thermally hydrosilylated with 1-dodecene to achieve a hydrophobic coating on the entire pSi surface. Following this, air plasma was utilized to remove the alkyl chains on the top surface, which was then further silanized with APTES. Following this process, the hydrophobic drug CPT was loaded into the hydrophobic pores through physical adsorption. Copolymers based on the monomer *N*-(2-hydroxypropyl) acrylamide (HPAm) and the photoactivatable cross-linker *N*-benzophenone acrylamide (BPAm) were prepared by RAFT polymerization. The resulting poly(HPAm-*co*-BPAm) polymer was then grafted to the APTES layer on the top layer of the modified pSi samples under UV irradiation. An increase in weight percentage of polymer in solution used for the spin coating process led to a corresponding increase in polymer thickness (as determined by ellipsometry on flat Si) (Fig. S1). For the 0.5 wt% sample, the average thickness was 33 nm. With the weight percentage increasing to 2 wt%, the average thickness increased to 98 nm.

**Figure 3:**
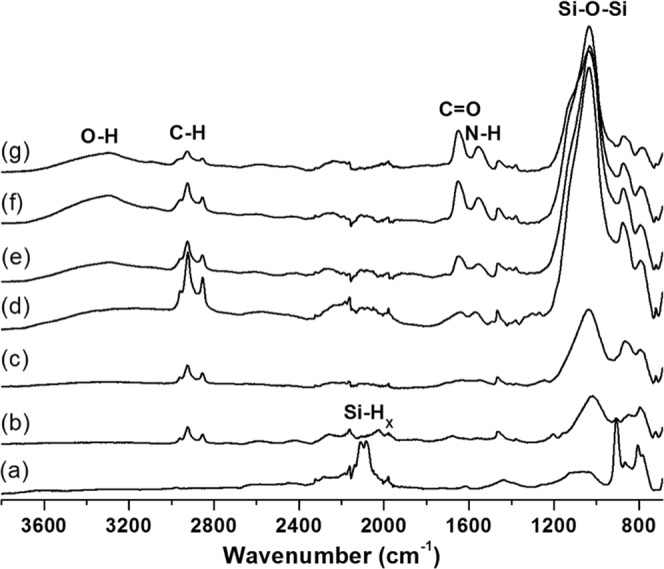
ATR-FTIR spectra of modified porous Si films with (**a**) freshly etched pSi, (**b**) pSi-dodecene, (**c**) pSi-dodecene-air plasma, (**d**) pSi-dodecene-air plasma-APTES and (**e**–**g**) pSi-dodecene-air plasma-APTES-0.5 wt%, 1 wt%, 2 wt% poly(HPAm-*co*-BPAm).

The surface chemistry was analyzed by ATR-FTIR spectroscopy. Figure 3 shows the IR spectra for pSi films at various stages during the chemical modification. The freshly etched pSi showed characteristic peaks at 2110 and 2083 cm^−1^ in spectrum (a), corresponding to the Si-H_2_ and Si-H stretching vibrations, respectively. After thermal hydrosilylation, the peak intensity of Si-H_x_ (x = 1, 2) decreased dramatically and the stretching vibrations of saturated C-H bonds in the 2850–2969 cm^−1^ range were observed. The IR spectrum showed no obvious difference after air plasma treatment to the pSi-dodecene films, except for the relatively stronger peak at 1035 cm^−1^ that corresponds to the Si-O-Si symmetrical stretching peak of silica. A set of peaks associated with amine stretching vibrational modes around 1568 cm^−1^ were apparent after silanization with APTES. The two broad bands in spectra (e-g) at 1652 and 1552 cm^−1^, assigned to C=O stretching and N-H bending vibrations, together with the O-H stretching band centered at 3302 cm^−1^, demonstrate the successful grafting of the polymer poly(HPAm-*co*-BPAm). With the polymer thickness increasing, the relative intensity between the amide and silica groups also increased.

Compared to freshly etched pSi (WCA = 64°), the pSi-dodecene samples exhibited much higher WCA (133°) (Fig. S2), indicating super hydrophobicity on the surfaces, as described previously^40^. The contact angle dramatically decreased to 11° after air plasma treatment and then increased to 78° after silanization with APTES. The poly(HPAm-*co*-BPAm) coated surfaces were determined to be more hydrophilic, with WCA values in the range of 50–55°. Those values are slightly higher than measurements obtained on HPAm hydrogels^39^, which may be attributed to the incorporation of hydrophobic *N*-benzophenone groups in the polymer chains or to the nanoscale topography of the pSi.

**Figure 4:**
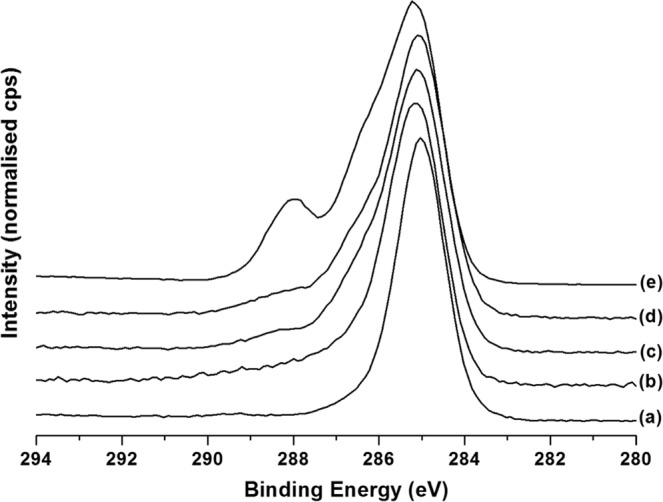
High-resolution XPS C 1 s spectra of (**a**) pSi-dodecene, (**b**) pSi-dodecene-air plasma, (**c**) pSi-dodecene-air plasma-APTES, (**d**) pSi-dodecene-air plasma-APTES-CPT, and (**e**) pSi-dodecene-air plasma-APTES-CPT-2 wt% poly(HPAm-*co*-BPAm). See text for details.

XPS analysis was also performed on surface modified pSi films. The XPS surface compositions are presented in Table 1, the corresponding carbon 1 s (C 1 s) high resolution spectra are displayed in Fig. 4. The hydrosilylated pSi sample showed predominantly carbon being present on the surface (68.6%), apart from Si and O (partially oxidized Si surface) and residual F from the HF etch. The corresponding C 1 s spectrum (Fig. 4a) consisted of a single, narrow and almost symmetric peak at a binding energy (BE) of 285 eV, consistent with dodecene (mainly CH_2_ moieties). Air plasma treatment removed most of the surface carbon (drop in C from 68.6% to 22.7%) with the exposure of a highly oxidized pSi surface (large increase in the O/Si ratio). The remaining carbon was also partially oxidized as is evidenced by the relative increase in C 1 s intensity at higher BE (286–290 eV, see Fig. 4b). Analysis of the pSi-dodecene-air plasma-APTES surface showed a slight increase in the C to 38.4% and a significant increase in nitrogen (N) to 7.4%, clear indication of the adsorption of APTES. This was further confirmed by the observed change in the C 1 s peak shape (Fig. 4c), the strong shoulder at 286–287 eV being consistent with C-O and C-N bonds in APTES. After drug loading no significant change in the surface composition was detected. Considering the very shallow sampling depth of XPS (5–10 nm) this suggests that most of the drug penetrated into pSi pores instead of adsorbing onto the outer surface. Likewise, the C 1 s peak shape was not affected by drug loading (Fig. 4d). Grafting of the surface with poly(HPAm-*co*-BPAm) resulted in a near elimination of the Si signal, indicating that a polymer layer with a thickness of probably greater than 10 nm was achieved. The surface composition consisted of C, N, and O only; the experimentally determined N:O ratio (0.53) was in close agreement with the theoretical value of 0.5 in the polymer. The successful grafting of poly(HPAm-*co*-BPAm) was further demonstrated by the presence of a strong C 1 s peak at 288.1 eV (Fig. 4e), consistent with C=O and N-C=O moieties in the polymer backbone.

**Table 1:** Summary of XPS elemental analysis of surface modified pSi samples. Surface compositions are expressed as atomic percentage (%).

surface	C	O	N	F	Si
pSi-dodecene	68.6	11.9	0.3	0.9	18.2
pSi-dodecene-air plasma	22.7	52.2	1.9	0.3	22.9
pSi-dodecene-air plasma-APTES	38.4	36.1	7.4	0.5	17.7
pSi-dodecene-air plasma-APTES-CPT	37.1	39.2	6.7	0.6	16.4
pSi-dodecene-air plasma-APTES-CPT-2 wt%	70.4	19.1	10.1	0.3	0.1

**Figure 5:**
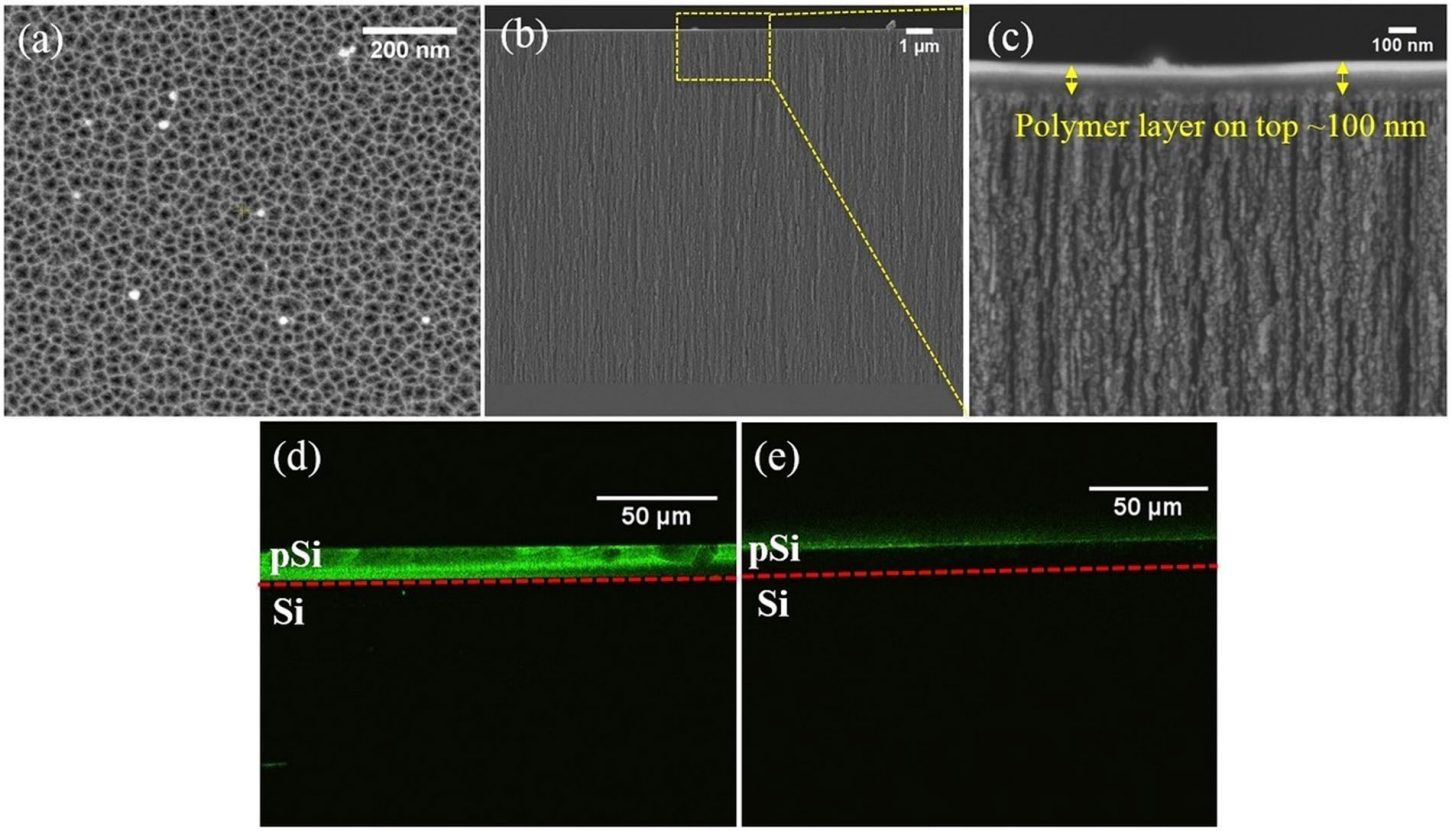
SEM images of (**a**) top-view of pSi (60 min), (**b**) cross-sectional view of pSi (60 min)-dodecene-air plasma-APTES-2 wt% poly(HPAm-*co*-BPAm), (**c**) higher magnification of (**b**). Confocal FITC images on cross section of (**d**) pSi (60 min)-ozone-APTES-FITC, (**e**) pSi (60 min)-dodecene-air plasma-APTES-FITC. The dashed red line represents the pSi-Si interface.

To facilitate confocal microscopy analysis, deeper pSi films were required. To produce these films, all etching conditions were kept the same except for the etching time which was increased from 5 min to 60 min, yielding cylindrical pores of approximately 20 nm in diameter (Fig. 5a) and 13 µm in depth (Fig. 5b). A cross-linked polymer layer (2 wt% poly(HPAm-*co*-BPAm)) was clearly observed on top of the pSi (Fig. 5c). The polymer thickness was approx. 100 nm, which was consistent with the value measured by ellipsometry. Confocal microscopy was used to confirm the effect of different surface modifications on samples that had been treated with FITC. Figure 5d shows the expected fluorescence signal for pSi (60 min)-ozone-APTES-FITC sample, in which APTES was applied all through the channels and reacted with FITC. In contrast, for pSi (60 min)-dodecene-air plasma-APTES-FITC sample, a sharp fluorescence signal only on top of the pSi layer was observed (Fig. 5e), confirming that APTES was only present on the exterior surface while the interior surface was coated with alkyl chains.

### pSi degradation kinetics

In order to determine the degradation rate of the pSi materials during the drug release process, the effective optical thickness (EOT) was monitored by time-lapse interferometric reflectance spectroscopy for 17 h as described in detail elsewhere^19^. The data summarized in Fig. S3 shows that oxidized pSi after silanization with APTES experienced a degradation rate of 0.935% EOT/h. This was considered fast with a loss of pore structure in a matter of 17 h. In contrast, the pSi-dodecene-air plasma-APTES sample was much more stable (0.022% EOT/h), showing almost no change of EOT over time. A similar behavior was also observed on cross-linked polymer capped pSi samples. The reason for the stable EOT is probably that both the porosity and medium (air or solvent filling the porous matrix) did not alter with polymer grafting^19^. Accordingly, any difference in the drug release profiles can be attributed to the properties of both interior and exterior surface chemistry rather than the degradation rate of the underlying pSi matrix except for the pSi-ozone-APTES samples.

### Drug release profiles

The release of the fluorescent cytotoxic drug CPT from the materials prepared in this study was investigated. CPT is a hydrophobic anticancer drug that affects human DNA topoisomerase І^41^. The loading capacity of CPT-loaded pSi films was determined after repeated sonication in PBS buffer until no more drugs was released and fitted to the calibration curve. Due to the relatively thinner films with smaller pore size prepared, the total amounts of CPT loaded into pSi-ozone-APTES and pSi-dodecene-air plasma-APTES (7.9 ± 3.5 and 16.0 ± 3.1 nmol cm^−2^, respectively) were lower than the amounts achieved in previous studies for CPT^6,19^. As expected, the hydrophobic surface chemistry resulted in an increase (doubling in this case) of the CPT loading capacity. The total amount of released CPT from 2 wt% polymer grafted pSi films (13.5 ± 2.5 nmol cm^−2^), was slightly lower than that from pSi-dodecene-air plasma-APTES samples. This was attributed to the loss of CPT during the spin coating process.

**Figure 6:**
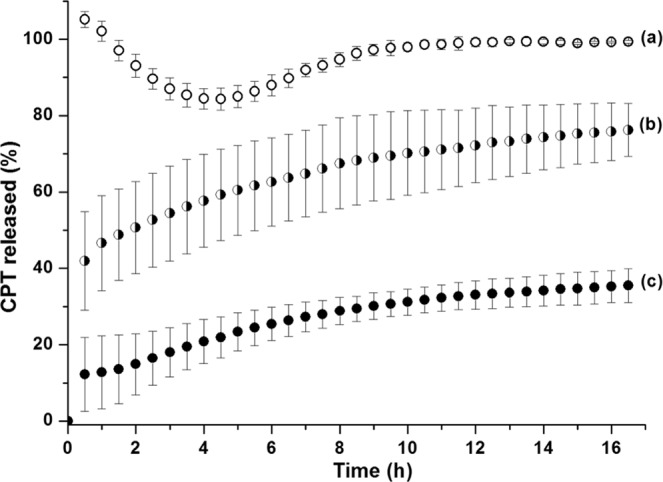
CPT release from (**a**) pSi-ozone-APTES, (**b**) pSi-dodecene-air plasma-APTES, (**c**) pSi-dodecene-air plasma-APTES-2 wt% poly(HPAm-*co*-BPAm).

The burst release feature of CPT drug from pSi-ozone-APTES samples was observed within the first 0.5 h (Fig. 6a). That might be attributed to the low affinity of CPT to the pSi pores and rapid diffusion from the pores. With CPT more dispersed in PBS solution, the CPT concentration started to decrease and reached equilibrium within 4 h. Then the samples continued to release CPT and completely released the drug in approx. 12 h. Similar release profiles were also observed in previous work^6,42^. Compared to the above samples, the release kinetics from pSi-dodecene-air plasma-APTES samples was much slower and showed sustained release over 17 h, although a burst release of 41.9% was still detected within the first half an hour (Fig. 6b), indicating that CPT drug molecules were more prone to be retained in the hydrophobic pSi channels. After 17 h, the average amount of CPT released from pSi-dodecene-air plasma-APTES was 76.2%. With polymer further grafted to the pSi outer surface, the burst release was only 12.2% within 0.5 h, followed by a slower and more sustained release of the entrapped CPT (Fig. 6c). This was expected since the poly(HPAm-*co*-BPAm) with abundant hydroxyl groups could form hydrogen bonds with water, thus generating a tightly bound hydration layer, which could slow down drug release, adding to the effect of the hydrophobicity of pSi inner pore surfaces.

### Protein adsorption on modified pSi surfaces

**Figure 7:**
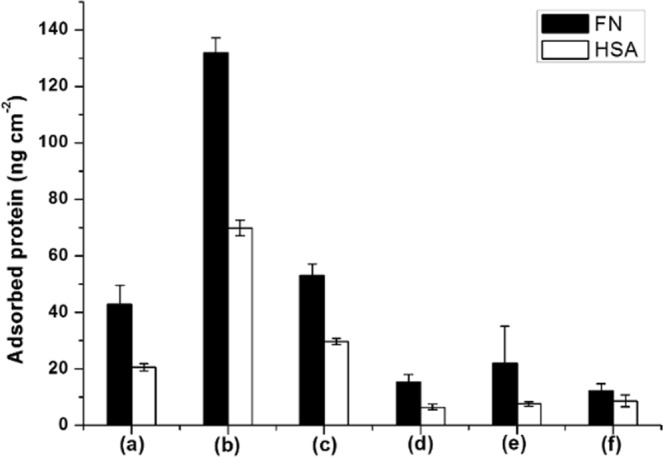
Eu-labelled fibronectin (FN) and human serum albumin (HSA) adsorption on different pSi surfaces with (**a**) pSi, (**b**) pSi-dodecene, (**c**) pSi-dodecene-air plasma-APTES, (**d**–**f**) pSi-dodecene-air plasma-APTES-0.5 wt%, 1 wt%, 2 wt% poly(HPAm-*co*-BPAm).

The spin coating and UV cross-linking technique were used to form polymer layers on the pSi outer surfaces in order to mediate the biological response at the bio-interface. Eu-labelled protein adsorption experiments were conducted to evaluate this response to modified pSi surfaces. Here, pSi-dodecene samples exposed to the Eu-labelled fibronectin (FN) solution resulted in a higher FN adsorption (132 ng cm^−2^) compared to bare pSi surfaces (43 ng cm^−2^) (Fig. 7), likely because the surfaces were more hydrophobic as shown in WCA measurements. A decrease to 53 ng cm^−2^ of FN adsorption was observed on pSi-dodecene-air plasma-APTES surfaces. With the capping of cross-linking polymers, the FN adsorption decreased further to less than 20 ng cm^−2^ which was slightly higher than that measured on HPAm polymer hydrogels^39^. A similar trend was also demonstrated in human serum albumin (HSA) adsorption experiments on pSi substrates. An obvious reduction in HSA adsorption (8 ng cm^−2^, 68% reduction relative to unmodified pSi and 90% reduction relative to pSi-dodecene surfaces) was observed for the pSi surfaces with the 0.5 wt% poly(HPAm-*co*-BPAm) coating. This value was even less than the minimum level achieved by *N, N’*-dimethylacrylamide (DMA) polymer grafting (25 ng cm^−2^)^43^. Taken together, these data indicate that the HPAm-based polymer coatings prepared by the UV cross-linking strategy effectively reduced protein adsorption on pSi surfaces.

### Inhibition of cell attachment on pSi surfaces

**Figure 8:**
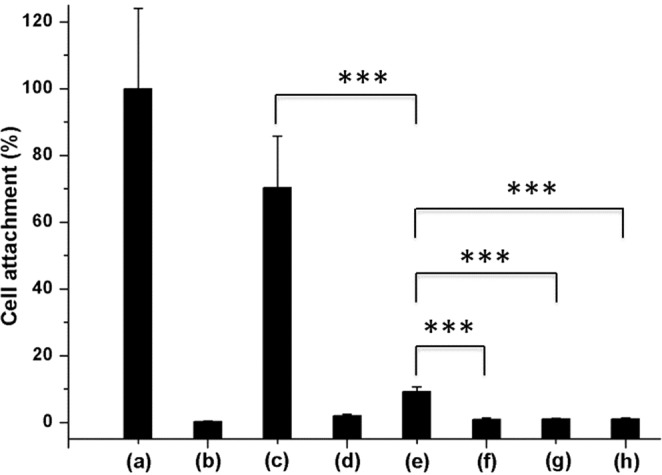
Cell counting on (**a**) tissue culture polystyrene (TCPS), (**b**) ultra-low attachment (ULA), (**c**) pSi, (**d**) pSi-dodecene, (**e**) pSi-dodecene-air plasma-APTES, (**f**–**h**) pSi-dodecene-air plasma-APTES-0.5 wt%, 1 wt%, 2 wt% poly(HPAm-*co*-BPAm) surfaces. (***p ≤ 0.001).

To investigate and quantify the cellular response to the different modifications on pSi surfaces, 24 h cell culture experiments were conducted using L929 mouse fibroblasts seeded in 24-well ultra-low attachment (ULA) plates with pSi substrates. Following a washing step to remove unbound cells, the remaining cells were fixed in paraformaldehyde and stained with DAPI to enable visualization of the nucleus. Representative images are shown in Fig. S4 (tissue culture polystyrene (TCPS) and ULA surfaces were chosen as positive and negative controls, respectively). The results obtained for each surface are shown relative to the readout obtained for the TCPS control surface (set to 100%). A substantial number of L929 cells (70% of TCPS) were observed on unmodified pSi surfaces (Fig. 8). However, pSi-dodecene samples were not cell-supportive because of their super hydrophobic character. L929 cell attachment on pSi-dodecene-air plasma-APTES surfaces was higher than for pSi-dodecene substrates. On the other hand, the presence of the poly(HPAm-*co*-BPAm) coatings (0.5 wt%, 1 wt% and 2 wt%) on pSi surfaces significantly reduced cell attachment (88–92% reduction) compared to pSi-dodecene-air plasma-APTES samples. No statistically significant difference was observed between any of the poly(HPAm-*co*-BPAm) surfaces or the commercial ULA surfaces. Overall, these results demonstrate that the cross-linked polymer used in this study is effective in reducing mammalian cell attachment to a minimum.

In conclusion, a nanostructured DDS was generated by functionalizing the interior and exterior surfaces of biocompatible pSi materials with different chemistries. The physicochemical characteristics of the fabricated pSi devices were investigated in detail. The hydrocarbon modified pSi matrix (pSi-dodecene-air plasma-APTES) showed improved (slower) camptothecin drug release kinetics compared to control samples (pSi-ozone-APTES). Using poly(HPAm-*co*-BPAm) as a capping layer, the drug release kinetics was even better controlled with a limited burst release (12.2% within first 0.5 h) followed by a slow release (32.3% after 17 h). Furthermore, the usage of a cross-linked HPAm based polymer on the pSi surfaces effectively reduced non-specific protein adsorption and cell attachment. This study provides insights into how to fabricate nanostructured DDS to meet multiple demands such as controlled release and non-fouling surface chemistry. The facile fabrication procedure provides a path for the future application of this surface modification strategy to pSi nanoparticles, with the aim of using them to fight cancer cells both *in vitro* and *in vivo*.

## Methods

### Materials and reagents

Si wafers were purchased from Siltronix, France. All chemicals (reagents and solvents) used for synthesis were purchased from Sigma-Aldrich at the highest purity available and used as received unless otherwise stated. For the Europium assay, reagents such as DELFIA enhancement solution and 100 nM Europium standard were obtained from PerkinElmer, Australia. Human serum albumin (HSA, 99%) and fibronectin (FN) were purchased from Sigma-Aldrich. For cell culture, the following reagents were used: paraformaldehyde (Sigma), DMEM medium (Invitrogen), fetal bovine serum (FBS, Invitrogen), Triton^™^ X-100 (Sigma) and 4’, 6-diamidino-2-phenylindole (DAPI) (Invitrogen), and were all used as received. L929 mouse fibroblast cells were used in cell culture experiments. *N*-(2-hydroxypropyl) acrylamide (HPAm) and *N*-benzophenone acrylamide (BPAm) were synthesized according to previous reports^39,44^.

### Preparation of pSi films

Nanostructured Si films were fabricated in a wet bench using 6 inch single-polished Si wafers (p++, 0.00055–0.001 Ω cm) in an electrochemical etching process. Si wafers were first electropolished at 22.7 mA cm^−2^ for 10 s in a solution of 1:1 (v/v) aqueous HF (48%): ethanol and the sacrificial layers dissolved using 1 M NaOH. Subsequently, pSi films were obtained by etching at a constant current density of 7.5 mA cm^−2^ for 5 min or 60 min. After etching, the samples were thoroughly rinsed with water and ethanol, and dried under a stream of nitrogen. pSi films were cut into 1 cm^2^ squares and kept in a desiccator for further usage.

### RAFT copolymerization of HPAm and BPAm

The initial feed molar ratio of BPAm to HPAm was 5 mol % to 95 mol %. A DMF solution of HPAm (1.84 M), BPAm (97.0 mM), 4-cyano-4-(((dodecylthio)carbonothioyl)thio)pentanoic acid (BM1432, 97%, Boron Molecular) (9.7 mM) and 2,2’-azobis(2,4-dimethyl valeronitrile (V65, 98%, Combi-B) (1.94 mM) was prepared in a nitrogen purged glove box. The solution was mixed at 65 °C for 100 min. After the polymerization, HPAm monomer conversion (90%) was determined by means of ^1^H NMR. Number-averaged molecular weight (Mn) and polydispersity (Mw/Mn) were determined by gel permeation chromatography (GPC) (Mn = 39500, Mw = 87000, PDI = 2.2). Copolymers were purified by reprecipitation with acetone. ^1^H NMR confirmed that 5 mol % of BPA was incorporated into the polymer chain. The RAFT polymer was described as poly(HPAm-*co*-BPAm).

### Surface modification of pSi films

Freshly etched pSi films were first hydrosilylated with 1-dodecene (96%, Alfa Aesar) at 135 °C for 2 h under a nitrogen atmosphere. The films were rinsed thoroughly with ethanol, then blown dry with nitrogen before being dried further in a desiccator overnight. The surfaces were then etched using an air plasma in a custom-built plasma reactor^45^. The input power was 20 W. Air was spiked into the vacuum chamber until the starting pressure reached 0.6 mbar, and a continuous radio frequency field was then generated between two electrodes for 10 s. Subsequently, the substrates were rinsed thoroughly with ethanol and silanization proceeded with 2% (3-aminopropyl)triethoxysilane (APTES) in anhydrous toluene for 10 min under gentle shaking^46^. The substrates were then washed with toluene and ethanol and dried under nitrogen, resulting in samples named *pSi-dodecene-air plasma-APTES*. For the preparation of control samples *pSi-ozone-APTES*, pSi films were oxidized in ozone atmosphere for 10 min, followed by silanization with APTES under the same conditions described above.

The RAFT polymer poly(HPAm-*co*-BPAm) was dissolved in ethanol with ratios of 0.5%, 1% and 2% (w/v). A 30 µl volume of each polymer solution was pipetted onto the previously prepared samples and spin-coated at 2000 rpm for 30 s. The samples were UV irradiated (power level 100%, Fusion UV Systems, Inc.) for 4 s to induce cross-linking. Substrates were then rinsed with ethanol to remove non-covalently attached polymers. The final samples were named *pSi-dodecene-air plasma-APTES-0.5* *wt%, 1* *wt%* and 2 *wt% poly* (*HPAm-co-BPAm*).

The samples for fluorescein isothiocyanate (FITC) conjugation were pSi films with 60 min etching time under the same etching current, namely *pSi (60* *min)-dodecene-air plasma-APTES*. *pSi (60* *min)-ozone-APTES samples* were chosen as control samples. The pSi substrates were incubated in 10 µg ml^−1^ FITC in ethanol for 1 h in the dark at room temperature and then washed thoroughly with ethanol. After drying, the samples named *pSi (60* *min)-ozone-APTES-FITC* and *pSi (60* *min)-dodecene-air plasma-APTES-FITC* were imaged immediately by means of confocal microscopy.

### Nuclear magnetic resonance (NMR) spectroscopy

NMR spectroscopy was performed using a 400 MHz Bruker spectrometer. ^1^H spectra were obtained using standard parameters with chemical shifts recorded in parts per million and referenced to the tetramethylsilane (TMS) peak at 0.00 ppm.

### Gel permeation chromatography (GPC)

GPC was performed using a Shimadzu system equipped with a CMB-20A controller system, a SIL-20A HT auto-sampler, a LC-20AT tandem pump system, a DGU-20A degasser unit, a CTO-20AC column oven, a RDI-10A refractive index detector and 4 Waters Styragel columns (HT2, HT3, HT4, HT5, each 300 × 7.8 mm^2^), providing an effective molar mass range of (100–4 × 10^6^). *N*, *N*-dimethylacetamide (DMAc) (containing 4.34 g L^−1^ lithium bromide (LiBr)) was used as an eluent with a flow rate of 1 ml min^−1^ at 80 °C. Number-average (Mn) and weight-average (Mw) molecular weights were evaluated using Shimadzu LC Solution software. The GPC columns were calibrated with low dispersity polystyrene (PSt) standards (Polymer Laboratories) ranging from 575 to 3,242,000 g mol^−1^. A third-order polynomial was used to fit the log M *versus* time calibration curve, which was linear across the molecular weight ranges.

### X-ray photoelectron spectroscopy (XPS)

XPS analysis was performed using an AXIS Nova spectrometer (Kratos Analytical Inc., Manchester, UK) with a monochromated Al Kα source at a power of 180 W (15 kV × 12 mA) and a hemispherical analyzer operating in the fixed analyzer transmission mode. The pressure during analysis was typically between 10^–9^ and 10^−8^ mbar. Survey spectra were collected at a pass energy of 160 eV. To acquire more detailed information, high-resolution spectra were recorded from individual peaks at 40 eV pass energy. Each specimen was analyzed at an emission angle of 0° as measured from the surface normal. Data processing was performed using CasaXPS software version 2.3.15 (Casa Software Ltd., Teignmouth, U.K.). Binding energies for all spectra were referenced to the aliphatic carbon peak at 285.0 eV. The atomic percentages of the detected elements were calculated using integral peak intensities and the sensitivity factors supplied by the manufacturer.

### Ellipsometry

The copolymer films were also prepared on flat Si surfaces and the thickness was measured with an ellipsometer (J.A. Woollam M-2000DI) using wavelengths between 250 and 1100 nm in 10 nm increments and at 65, 70 and 75° from the surface normal. To obtain the ellipsometric thickness of the grafted films, the VASE spectra were fitted with the multilayer model using the WVASE32 analysis software, and the optical properties of a generalized Cauchy layer.

### Water contact angle (WCA) measurements

The WCA of surfaces used in this study were measured using an optical tensiometer (KSV CAM 200) at 25 °C. A ~3 μl drop of Milli-Q water was carefully placed on the surface of a dry sample. Images were captured and WCA values were automatically calculated from images by the software. All measurements were repeated at least three times and the results were averaged.

### Scanning electron microscopy (SEM)

The samples were mounted on aluminium stubs. These samples were then iridium coated using a Cressington 208HRD sputter coater. The thickness of the iridium coating was approximately 4 nm (60 mA for 50 seconds). The samples were imaged using a Zeiss Merlin FESEM (Field Emission Scanning Electron Microscope) operated in the secondary electron (SE) mode. The images were obtained using 2 kV and a probe current of 120 pA.

### Attenuated total reflection-Fourier transform infrared (ATR-FTIR) spectroscopy

ATR-FTIR spectra were recorded using a Nicolet 6700 (Thermo Scientific) instrument coupled to a diamond detector. The spectra collected were averages of 64 scans recorded with a resolution of 4 cm^−1^. Background spectra were blanked using air. The data was processed using OMNIC software.

### Interferometric reflectance spectroscopy (IRS)

The effective optical thickness (EOT) of the pSi layer was measured via IRS in time-lapse mode. This equipment was described in detail elsewhere^47^. Briefly, a custom-built interferometer operating with a S2000 CCD Detector (Ocean Optics, USA) was used to capture the reflectivity spectra from pSi substrates which were placed inside a custom-built fluidic cell. The PBS solution was allowed to flow over the sample at 20 µl min^−1^. The change of EOT was recorded for 17 h.

### Camptothecin (CPT) loading and release

CPT loading was performed by using a cylindrical stainless holder with a rubber ring underneath which could firmly attached on the sample surfaces (surface area 0.5 cm^2^). A volume of 100 µl of a 2.5 mg ml^−1^ CPT in DMF was dropped into the holder and the incubation was allowed to proceed at room temperature in the dark for 2 h. Samples pSi-ozone-APTES (as control) and pSi-dodecene-air plasma-APTES were subjected to the same loading procedure described above. After loading, the substrates were quickly dipped in water three times and acetone once to remove excess loosely attached drug, followed by overnight drying in a desiccator under aluminium foil protection.

The release of CPT from loaded samples was monitored using a fluorimeter according to our previously reported method^19^. Drug release measurements were performed using a luminescence spectrometer (LS55, PerkinElmer) fitted with a temperature control system (Peltier, T = 37 °C). The quartz cuvette was filled with 3 ml phosphate buffered saline (PBS) (pH = 7.4), and the CPT-loaded sample was immersed into it and placed outside of the optical path. The release of the drug over 17 h was recorded with an excitation wavelength of 370 nm and an emission wavelength of 434 nm (slit width = 3 nm) under sink conditions. Release intensities of CPT were converted to amounts using a calibration curve and then normalized to the surface area. The total loading amount of drug was determined by sonicating samples over 8 h and used to convert the amount of release to a percentage. No fewer than three release curves for each sample were averaged.

### Europium (Eu)-labelled protein adsorption studies

Eu-labelled protein adsorption studies were carried out as described in a previous report^43^. Unlabelled HSA solution (5,000 µg ml^−1^) and Eu-HSA (2.8 µg ml^−1^) as well as unlabelled FN (5,000 µg ml^−1^) and Eu-FN (35.3 µg ml^−1^) were mixed in 1000:1 and 100:1 ratios (by protein mass), respectively. The prepared protein solutions (50 µl) were then added onto substrates and incubated for 1 h under gentle shaking in the dark at 37 °C using a hot plate. Then the Eu-tagged protein solution was carefully removed and substrates were dipped in PBS five times and completely dried with nitrogen. Enhancement solution was then pipetted onto the substrates and incubation proceeded in the dark for 45 min at room temperature to release the Eu-complex. Aliquots (100 µl) of Eu-complex solution were transferred to 384-well black plate and read on the PHERAstar instrument (BMG LABTECH Pty. Ltd.) using a time-resolved fluorescence assay. A calibration curve was prepared by measuring the fluorescence intensities of 0.005 nM to 1 nM dilutions of Eu standard in PBS. The data obtained from the plate reading were then fitted to the calibration curve. Protein adsorption results were averaged in four replicate samples, expressed as adsorbed protein amount (ng cm^−2^).

### Cell culture experiments

All samples were placed in 24 well ultra-low attachment plates (ULA, Corning) and incubated in 80% ethanol for 30 min, followed by incubation with 2 × antibiotic antimycotic solution (anti-anti, Gibco) in PBS for 1 h to minimize the risk of microbial and fungal contamination. Following this, the anti-anti solution was removed and the samples were dried and sterilized under UV radiation in a sterile cabinet for 1 h. L929 mouse fibroblast cells were added onto each sample at a density of 10000 cells cm^−2^ in media containing 10% fetal bovine serum (FBS) and 1 × anti-anti. After 24 h incubation at 37 °C 5% CO_2_, the cells were fixed in a 4% paraformaldehyde solution for 20 min at room temperature. The cell membranes were permeabilized by adding 0.1% Triton-100X surfactant for 20 min. Subsequently, cell nuclei were stained with DAPI in PBS. Images of the sample surfaces were obtained using a Nikon Eclipse TE2000-U microscope. Cells were also seeded on tissue culture polystyrene (TCPS, Nunclon delta surface, Nunc) and ULA control surfaces under the same conditions. Cell counting was conducted from four represented fluorescence microscopy images obtained on each modified Si surfaces (triplicate).

## Supplementary information

Spatially Controlled Surface Modification of Porous Silicon for Sustained Drug Delivery Applications
