# Supplementary material for: Spatially Controlled Surface Modification of Porous Silicon for Sustained Drug Delivery Applications
Source: Sci Rep. 2019 Feb 4;9:1367. doi: 10.1038/s41598-018-37750-w (PMC6361965; doi:10.1038/s41598-018-37750-w)
Supplement: Supplementary file 1 — Spatially Controlled Surface Modification of Porous Silicon for Sustained Drug Delivery Applications [file 41598_2018_37750_MOESM1_ESM.docx]

Supplementary Information

**Spatially Controlled Surface Modification of Porous Silicon for Sustained Drug Delivery Applications**

# De-Xiang Zhang1,2, Chiaki Yoshikawa2,3, Nicholas G. Welch2, Paul Pasic2, Helmut Thissen2,*, Nicolas H. Voelcker1,2,4,*

1Drug Delivery, Disposition and Dynamics, Monash Institute of Pharmaceutical Sciences, Monash University, Parkville, Victoria, 3052, Australia

2Commonwealth Scientific and Industrial Research Organisation (CSIRO) Manufacturing, Clayton, Victoria, 3168, Australia

3International Centre for Materials Nanoarchitectonics, National Institute for Materials Science, 1-2-1, Sengen, Tsukuba, Ibaraki, 305-0047, Japan

4Melbourne Centre for Nanofabrication, Victorian Node of Australian National Fabrication Facility, Clayton, Victoria, 3168, Australia

*E-mail: [helmut.thissen@csiro.au](mailto:helmut.thissen@csiro.au)

*E-mail: [nicolas.voelcker@monash.edu](mailto:nicolas.voelcker@monash.edu)

**Results**


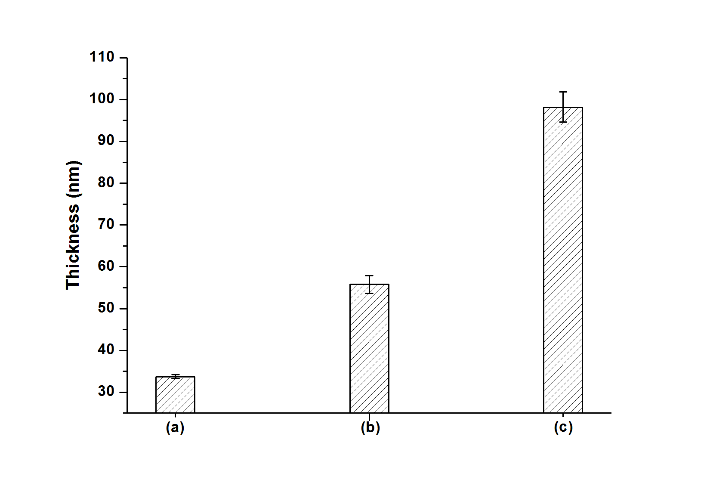


**Figure S1.** RAFT polymer thickness measurement on flat Si surfaces (a-c) Si-ozone-APTES-0.5 wt%, 1 wt%, 2 wt% poly(HPAm-*co*-BPAm).


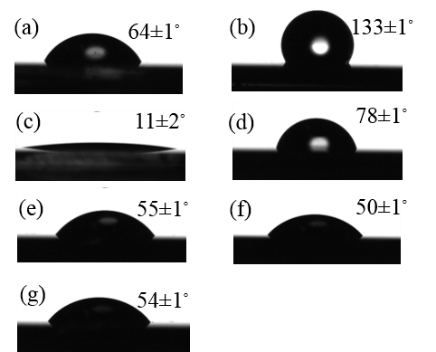


**Figure S2.** Water contact angle (WCA) of modified porous Si films (a) freshly etched pSi, (b) pSi-dodecene, (c) pSi-dodecene-air plasma, (d) pSi-dodecene-air plasma-APTES, (e-g) pSi-dodecene-air plasma-APTES-0.5 wt%, 1 wt%, 2 wt% poly(HPAm-*co*-BPAm).


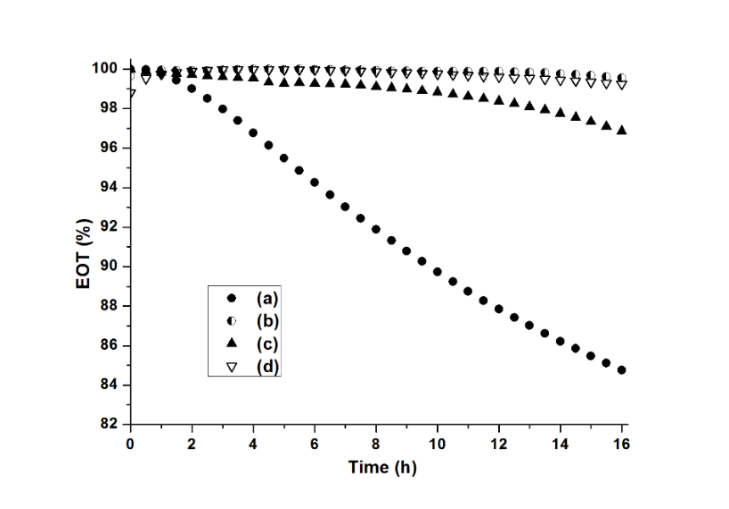


**Figure S3.** Time-lapse effective optical thickness (EOT) measurements for (a) pSi-ozone-APTES, (b) pSi-dodecene-air plasma-APTES, (c-d) pSi-dodecene-air plasma-APTES-0.5 wt%, 1 wt% poly(HPAm-co-BPAm).


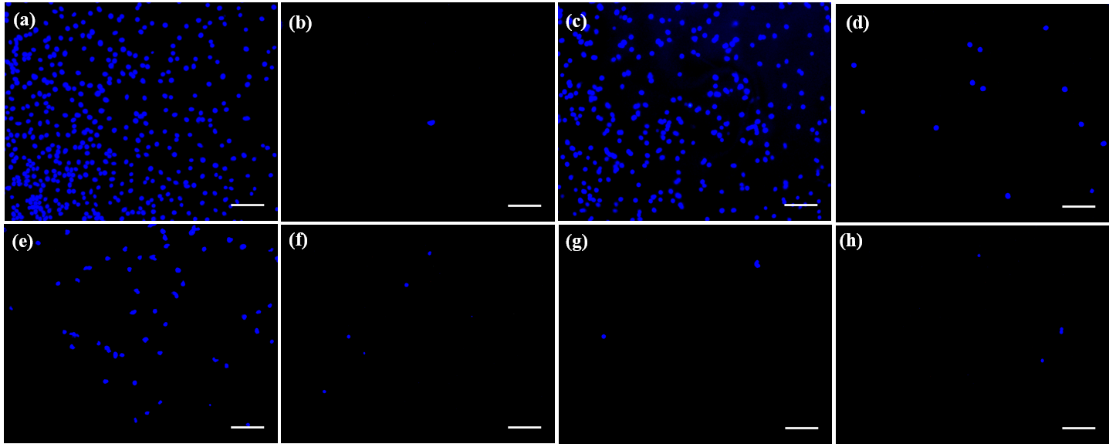


**Figure S4.** Represented fluorescence microscopy images of L929 mouse fibroblasts after 24 h culture on (a) tissue culture treated polystyrene (TCPS), (b) ultra-low attachment (ULA), (c) pSi, (d) pSi-dodecene, (e) pSi-dodecene-air plasma-APTES, (f-h) pSi-dodecene-air plasma-APTES-0.5 wt%, 1 wt%, 2 wt% poly(HPAm-*co*-BPAm) surfaces. Cells were fixed in paraformaldehyde solution and stained with DAPI for the nucleus (blue). Scale bars represent 200 µm.
